# Notes on Mummification of the Dental Pulp

**Published:** 1903-04

**Authors:** Walter H. Neall

**Affiliations:** Philadelphia


### NOTES ON MUMMIFICATION OF THE DENTAL PULP.[1]

BY WALTER H. NEALL, D.D.S., PHILADELPHIA.

THE assertion can be safely made, without fear of contradiction, that there is not a dentist in active practice but who has had his share of the bother, discomfort, and annoyance of treating pulps in some stage of disorder.

Now, if the treatment could be reduced to an exact science, an infallible rule laid down which, when followed out, would give positive results, pulp treatment would be regarded as a luxury, at least so far as the dentist is concerned, as well as being the greatest possible benefit to the patient of lax nerve. For accomplishing this desired end mummification of the dental pulp has made a bid for recognition.

The question naturally arises, Is the pulp really mummified, or is it subjected to a long, lingering death without pain? or again, Is it preserved and held in bondage to awaken into life, activity, and decay upon its release from its enthralment?

Several dentists have asserted that months after the said mummification was supposed to have taken place sensation and positive pain had been experienced in the course of preparing the tooth for filling. Others have found the pulp in exactly the same condition it originally was, when they have uncovered it for the purpose of examination, and others have observed a peculiar leathery appearance of this vital organ, to be spoken of later in connection with attendant suffering.

Mummification is the direct opposite to the long-practised method of capping the pulp. In pulp-capping the desire is to pre-

---

[1] Read before the Academy of Stomatology, October 28, 1902.

serve it in its living state; in the mummifying method the object is to destroy it and embalm it within its bony walls.

As practised by the ancients, mummification is a lost art; embalming, as used at the present time, is not lasting. It must be considered that the ancients spent many days in the employment of the mummifying chemicals upon the dead body and that parts subject to putrefaction were carefully removed; then, again, absolute dryness played an important part; the cavity was peculiarly filled in this respect.

It is doubtful whether the bodies of the Egyptians could have been so well preserved if moisture had had access to them. In pulp mummification, if one could keep out moisture,—that is, the accumulations at the apical end of the root of the tooth under treatment,—the dentist's difficulties would be reduced to a very small compass; but it is the lack of being able to control moisture that works the mischief, and therefore it is reasonable to presume that a pulp cannot be absolutely mummified or embalmed.

It seems to be the consensus of opinion that the best results are obtained when as much as possible of the pulp is removed by the aid of the cocaine pressure method or in the employment of an arsenical preparation, that which remains in the canal being treated with the mummifying material. The writer claims that this is not mummification in the strict acceptance of the term; it is simply employing a method of treatment that has been known and practised for years, the difference being in the medicaments used.

Conservative dentists are deterred in the use of a mummifying agent to the dental pulp *in situ* because the answer to "What will the harvest be?" is enveloped in too many painful and complex possibilities.

A brief history of some twenty-one cases, covering a period of a little over a year, is interesting. In every instance the application of the mummifying compound was made in strict accordance with the directions. Of the material used, one was a patent preparation, ingredients vague; another, a paste procured at a dental supply depot, ingredients published; and another a mixture made for the writer, employing a formula of long use. It is, of course, presumed that the drugs comprising a mummifying paste are known and that the manner of applying the same is understood; going into details would consume too much time in a ten-minute paper.

Of these twenty-one cases, nine were persons presenting themselves with either exposed or inflamed pulps; five were persons who do not visit a dentist until their teeth begin to trouble; of the remainder, the cause of distress was equally divided between the rapid decay of the third molar, neglect owing to ill health, and the frequent tooth disturbances due to pregnancy. It is well to state that all of these cases have been communicated with during the past month.

Cases 1 and 12.—Adult female (two operations in one month). Treatments were in the first and second molars, left superior jaw. Bulbous portion of pulp removed; balance, very tender at that, mummified. Up to the present time perfectly comfortable.

Case 2.—Adult female. Right inferior first molar; anterior root curved; impossible to extract pulp. No discomfort.

Cases 3 and 9.—Adult female (two operations in one month). One operation was upon the right superior first molar, the other upon the left superior first molar. These teeth erupted at the time of a severe illness; in consequence their crowns were bereft of protecting enamel. Operation on the third case was performed in May, 1901, the complete pulp being treated to the mummifying material. Quiet reigned until November of this year, when the tooth became sore and ached violently. Upon the filling, etc., being removed, the pain ceased. The pulp was found in a peculiarly leathery condition; not hard and flint-like, but tough, yet not easily penetrated with a sharp explorer. The tooth has been re-treated and filled.

*Query:* Could pulp capping have produced any worse results?

The ninth case, being in the same mouth, was treated at about the same time; the buccal roots were the ones involved. These have given no uneasiness.

Case 4.—Adult female. An extremely loose superior molar, almost on the verge of dropping out; much drilling and manipulation would surely have caused its loss. Pulp treated to mummifying paste. Reported to be "in splendid condition."

Case 5.—Adult female. A left superior third molar; bulbous portion of pulp removed; balance treated to the preserving medicament. Excellent result.

Case 6.—Adult male. Left superior first molar; could not devote time to lengthy treatment; portion of pulp removed, balance mummified. Report, in good condition.

Case 7.—Adult male. Right superior wisdom-tooth, much broken down; would not listen to extraction; pulp mummified as a whole. Condition excellent.

CASE 8.—Adult male. A stubborn right superior first bicuspid; impossible to remove all the pulp from canal; mummification resorted to. Success pronounced.

CASE 10.—Minor; male. An exasperating patient, irritable, nervous, and disobedient. Tooth, the left superior first bicuspid, presented a lingual decay, embracing exposure of the pulp. Weeks were spent on the case, a cotton dressing hardly being tolerated. With fear and trembling on the dentist's part, mummification was resorted to and the tooth permanently filled. Not a vestige of pain or discomfort has been experienced since.

CASE 11.—Adult female. A broken-down left inferior lateral; part of pulp removed, balance mummified. Excellent result.

CASE 13.—Adult female. An elongated left superior second molar; decay reaching pulp-cavity through the root; removed as much as possible of bulbous portion of the pulp; treated balance to mummification. Reported as having no pain or annoyance.

CASE 14.—Adult female; monthly nurse. Right inferior first molar; no time for treatment; removed part of pulp, mummified balance; patient immediately left city. Reported comfortable.

CASE 15.—Adult female. A person who always wishes things done in a hurry; had no time and does not care to bother. Tooth, left superior second molar; decayed to exposure on distal aspect; very little of pulp removed; balance treated as in former cases. No discomfort has resulted.

CASE 16.—Adult female, much run down in health and a corresponding weakness of the teeth. The right inferior first bicuspid was deprived of part of its pulp, and the exceedingly sensitive balance was mummified. Reports no trouble or inconvenience.

CASES 17 AND 18.—Adults, male and female, a third molar in each case being involved. Both buccal decays; there were no extractions of the pulp. Both reported a short time ago in excellent condition.

CASE 19.—Adult female. Right inferior first molar; pulp in anterior root, being treated to the mummifying process, remains in quiet and comfort.

CASES 20 AND 21.—Adult females. First molars in each case; as much of pulps removed as possible, the balance being subjected to mummification; each case developed, within a day or two, the most violent pains, necessitating the immediate removal of the filling and the paste. The teeth quickly responded to treatment, however, and at the present time are in a quiescent state, still unfilled.

The average success observed in the cases just cited seems to warrant a continued and frequent use of a mummifying material in conjunction with pulp treatment, but the results obtained by the writer are far from convincing, for there may yet be a day of reckoning.

